# Estudio comparativo de diferentes métodos de análisis de validez de la muestra para la detección de drogas en orina: análisis de pH y densidad, tira de detección de adulterantes TECO™ y análisis de oxidantes

**DOI:** 10.1515/almed-2021-0052

**Published:** 2021-08-11

**Authors:** Ashraf Mina, John Stathopoulos, Taveet Sinanian, Leah McNeice, Deirdre Holmes, Kristi-Lee Fletcher, Emily Bottero, Shanmugam Banukumar, Santiago Vazquez

**Affiliations:** Servicio de Medicina Forense y Analítica (FASS), Unidad de Toxicología, NSW Health Pathology, Hospital Macquarie, Sydney, NSW, Australia; Facultad de Medicina y Ciencias de la Salud, Universidad de Sidney, Sydney, NSW, Australia

**Keywords:** adulteración por fármacos, análisis de oxidantes, gravedad específica, pH, validez de la muestra

## Abstract

**Objetivos:**

La adulteración de una muestra de orina por parte de un paciente puede pasar inadvertida si no se realiza una prueba de validez de la muestra. En este estudio se ha comparado el método análisis de densidad (SG, según acrónimo inglés), pH, la tira para detección de adulterantes TECO™ (Dipstick) y el análisis de oxidantes, para determinar las diferencias entre los mismos y permitir una toma de decisiones informada, a la hora de seleccionar un método.

**Métodos:**

El análisis de la creatinina, la SG y el pH es esencial a la hora de realizar una prueba de validez de la muestra. Se compararon los métodos químicos automáticos de análisis de SG y pH con la potenciometría para el análisis de pH, la refractometría para el análisis de SG, y el método dipstick. Así mismo, se realizó una comparación entre el análisis de oxidantes y el método Dipstick.

**Resultados:**

Se observó una concordancia del 81,9% y del 64,7% entre el método químico de análisis de SG y la refractometría y el método Dipstick, respectivamente. La concordancia entre la refractometría y Dipstick fue del 66,1%. La concordancia entre el método químico de análisis del pH y la potenciometría y el método Dipstick fue del 74,3% y del 81,4%, respectivamente. La concordancia entre la potenciometría y el método Dipstick fue del 85,7%. El análisis de resultados se realizó mediante el método de regresión de Deming y la prueba F. Los resultados demuestran una mejor correlación entre el método químico de análisis de SG y la refractometría, que con el método Dipstick. El análisis de oxidantes mostró una buena correlación con Dipstick a la hora de detectar adulterantes como el clorocromato de piridinio, el nitrito y la lejía.

**Conclusiones:**

Se observaron diferencias en la medición de SG y pH. Dichas diferencias se deben tanto a factores metodológicos como instrumentales. Se recomienda el empleo de métodos químicos automatizados, así como del análisis de oxidantes debido a sus consistencia, precisión y mejor tiempo de respuesta para realizar la prueba de validez de la muestra para detectar la presencia de drogas.

## Introducción

Las pruebas de detección de drogas en orina se realizan en la medicina de urgencias, la gestión de prescripciones y las pruebas de detección de drogas en el lugar de trabajo, así como en el derecho penal. Algunas drogas ilícitas son las anfetaminas, el éxtasis, la cocaína, las benzodiazepinas, los cannabinoides (marihuana), los opiáceos, la heroína, la oxicodona, los alucinógenos, los inhalantes, el fentanilo o el uso no médico de psicoterapéuticos recetados, como los analgésicos, tranquilizantes, estimulantes y sedantes recetados. Las pruebas de drogas poseen implicaciones personales, profesionales y jurídicas, y los médicos de atención primaria deben saber interpretar los resultados de las pruebas de drogas en orina para optimizar los resultados clínicos [1]. Algunos pacientes tratan de invalidar las pruebas de drogas en orina ingiriendo agentes de desintoxicación o lavado, diluyéndolas en agua y otros líquidos y/o adulterándola con otras sustancias como la lejía o el nitrito, o sustituyendo las muestras con orina humana libre de drogas o con orina sintética [2].

La adherencia se puede enmascarar con orina diluida, productos de limpieza, aditivos de la orina, la cantidad de droga consumida, el tiempo transcurrido desde la última dosis, la sustitución de la muestra de orina, la sustitución con orina sintética, los puntos de corte empleados en el laboratorio y la incertidumbre de la medición (MU). Un resultado negativo en una muestra de orina diluida puede llevar a una interpretación errónea sobre el consumo de drogas de un sujeto. La Administración de Servicios de Salud Mental y Abuso de Sustancias (SAMHSA, según el acrónimo inglés) de los EE.UU, establece como obligatorio analizar la densidad (SG) y el pH de las muestras de orina para verificar la validez de la muestra [3].

Así mismo, se recomiendan otros protocolos para detectar la adulteración de la orina [4], [5], [6], como la medición de temperatura, y el análisis de creatinina, SG, oxidantes y pH.

La orina normal fresca tiene una temperatura de entre 32,5 y 37,7 °C, pH entre 4,7 y 7,8 [7, 8], SG entre 1,003 y 1,035 g/mL [7, 9] y concentración de creatinina entre 80 y 200 mg/dL (7,07–17,68 mmol/L) [9, 10]. Si el valor de alguno de estos parámetros queda fuera de los rangos de normalidad, la muestra de orina podría haber sido adulterada.

El objetivo de este estudio es comparar diferentes métodos de determinación de pH y SG empleando el analizador químico Beckman-Coulter AU5810, un potenciómetro, un refractómetro para SG y Dipstick.

Se evaluó también la interferencia de clorocromato de piridinio, nitrito, nitrato, e hipoclorito de sodio mediante el análisis de oxidantes.

De los resultados obtenidos se beneficiarán aquellos pacientes que se vayan a someter a una prueba de detección de drogas u otra evaluación clínica que incluya dicha prueba. Comparamos el rendimiento de seis métodos de determinación de pH y SG. También se ha comparado el rendimiento del análisis de oxidantes frente a Dipstick. Esto permitirá a los laboratorios evaluar los métodos que emplean y conocer las variaciones entre ellos, facilitando la elección informada del método a emplear.

## Materiales y métodos

El pH y la SG se determinaron con el analizador Beckman-Coulter AU5810. Se emplearon los siguientes reactivos químicos de Thermo Fisher: prueba de pH (CDF100054), calibrador de pH, (CDF100283), control de pH 7 (CDF100284) y control de pH 10 (CDF100285), prueba de gravedad (CDF1194), calibrador de densidad específica baja (CDF1754), calibrador de densidad alta (CDF1755), control de densidad de nivel 1 (CDF1756), control de densidad de nivel 2 (CDF1757) y el análisis de oxidantes de Thermo Fisher (10009958).

El pH se midió con el medidor de pH/iones Mettler Toledo Seven Compact™, mientras que se empleó el refractómetro digital PA202 de Micro Palm Abbe para la SG.

Las interferencias se detectaron empleando los siguientes reactivos: blanco de orina certificada de PM Separation (88121-CDF-L UTAK), material de referencia certificado de nitrito de Choice Analytical, (IC-N-M-100), lejía (hipoclorito de sodio) 125 g/L de Jasol (2066070), y clorocromato de piridinio al 98% de Aldrich (19.014-4). Para la determinación de creatinina, pH, SG, nitrito, lejía, clorocromato de piridinio y glutaraldehído se utilizaron las tiras de detección de adulterantes TECO de Techo Diagnostics (CDA700-25).

En este estudio se emplearon las muestras de orina habitualmente analizadas en nuestro laboratorio, procedentes de hospitales, centros de menores y clínicas. Para la determinación de la SG, se analizaron 204 muestras con el método químico de Thermo Fisher en el analizador Beckman-Coulter AU5810, el refractómetro y Dipstick. Para el pH, se analizaron 210 muestras con el método de Thermo Fisher en el analizador Beckman-Coulter AU5810, el potenciómetro y Dipstick. Se analizaron partes alícuotas en distintas estaciones de trabajo a lo largo de una semana. Los resultados se analizaron mediante el análisis de regresión ponderada de Deming y la prueba F. Así mismo, se calculó la concordancia (%) entre los métodos en base al número de resultados que quedaban dentro o fuera del intervalo de referencia.

Se realizaron diluciones seriadas de nitrito, nitrato, lejía (hipoclorito de sodio) y clorocromato de piridinio en orina limpia para imitar la orina adulterada. Dichas diluciones se analizaron con el análisis de oxidantes y el método Dipstick para compararlos. Dado que el nitrito es endógeno en la orina y también se puede emplear como adulterante, se analizó vertiendo nitrito de sodio de laboratorio en blanco de orina certificada, empleando también material de referencia de nitrito certificado (CRM). Para la preparación de las diluciones seriadas se empleó blanco de orina certificada el primer día de preparación. Tras el análisis inicial de las diluciones, las muestras se volvieron a analizar 17 días después para comprobar la estabilidad de los adulterantes en la orina.

El presente estudio está exento de aprobación por parte de un Comité Ético, ya que no implica a humanos o animales. Además, la prueba de validez de la muestra se realiza de manera ordinaria en el laboratorio.

## Resultados

Para la regresión de Deming se emplearon medidas emparejadas (*x*_*i*_, *y*_*i*_), medidas con errores (*ε*_*i*_ y *δ*_*i*_), donde *x*_*i*_ = *X*_*i*_ + *ε*_*i*_ y *y*_*i*_ = *Y*_*i*_ + *δ*_*i*_, para calcular la ordenada en el origen (*β*_0_), y la pendiente (*β*_1_), en la ecuación $\bar{Y}{\bar{Y}}_{i}=\beta_{0}+\beta_{1}\bar{X}{\bar{X}}_{i}$ , donde $\bar{X}{\bar{X}}_{i}$ y $\bar{Y}{\bar{Y}}_{i}$ son estimaciones del valor “observado” (o predicho) de *X*_*i*_ y *Y*_*i*_, respectivamente. La regresión de Dening se emplea para la comparación de métodos en el campo de la química clínica para buscar diferencias sistemáticas entre dos métodos de medida. La pendiente muestra el sesgo proporcional, que suele relacionarse con diferencias de calibración entre métodos, mientras que la ordenada en el origen muestra un sesgo constante y puede relacionarse con aspectos de calibración y valor de consigna. La distribución de F es el cociente de dos varianzas o, técnicamente, dos medias cuadráticas, y se emplea para averiguar si existen diferencias significativas entres los valores medios de dos poblaciones. En la hipótesis nula, la distribución de F es aproximadamente de 1. Para rechazar la hipótesis nula de que las medias son iguales en los dos grupos, se necesita un valor de F elevado.

**Figura 1:**
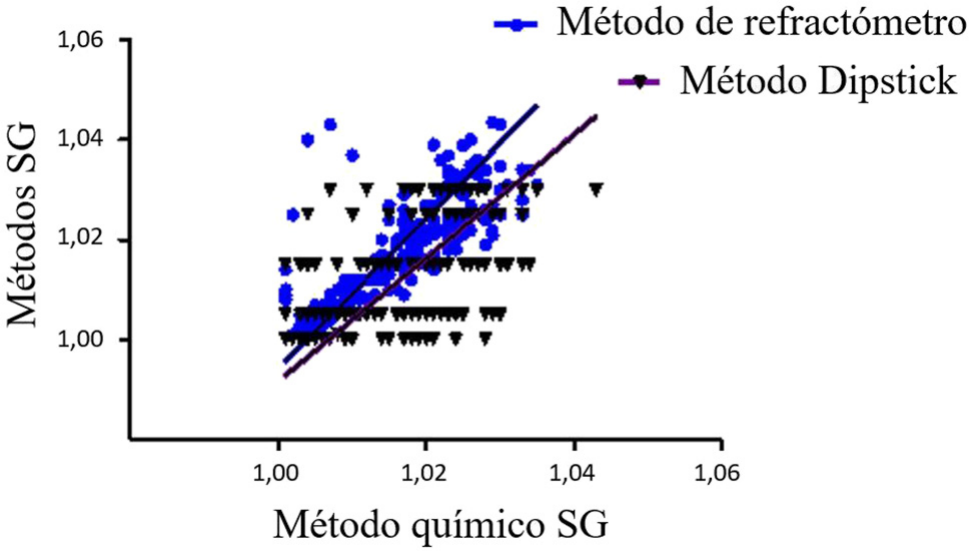
Análisis de regresión de Deming del método químico de análisis de GE de Thermo Fisher frente al refratómetro y la tira de detección de adulterantes en orina de TECO (Dipstick).

El análisis de datos se realizó mediante la regresión ponderada de Deming y la prueba de F (Figura 1 y Tabla 1). Se observó una diferencia estadísticamente significativa entre el refractómetro de SG y el método Dipstick de SG, y el método químico de Thermo Fisher. El refractómetro de SG mostró mayor sesgo proporcional y constante que el método Dipstick de SG, comparado con el método de Thermo Fisher.

**Tabla 1:** Regresión de Deming y prueba de *F*-test para: (A) Análisis químico de análisis de GE de Thermo Fisher frente a la refractometría y la Tira de detección de adulterantes en orina de TECO (Dipstick). (B) Análisis químico de análisis de pH de Thermo Fisher frente al potenciómetro y a la Tira de detección de adulterantes en orina de TECO (Dipstick).

A) Análisis de regresión de Deming	GE según el método de refractometría	GE según Tira de detección de adulterantes en orina TECO (Dipstick)
Ecuación	${\bar{Y}}_{i}=\beta_{0}+\beta_{1}{\bar{X}}_{i}$	${\bar{Y}}_{i}=\beta_{0}+\beta_{1}{\bar{X}}_{i}$
Ordenada en el origen (*β*_0_). (IC 95%)	−0,5092 (entre −0,6847 y −0,3337)	−0,2430 (entre −0,5139 y 0,02786)
Pendiente (*β*_1_). (IC 95%)	1,5000 (entre 1,330 y 1,676)	1,2345 (entre 0,9679 y 1,501)
**A) Prueba de F**	293,9	83,25
DFn	202,0	202,0
Valor de P	<0,0001	<0,0001
Desviación de cero?	Significativa	Significativa
Número total de valores	204	204

**B) Análisis de regresión de Deming**	**pH según el método de potenciometría**	**pH según la Tira de detección de adulterantes en orina TECO (Dipstick)**

Ecuación	${\bar{Y}}_{i}=\beta_{0}+\beta_{1}{\bar{X}}_{i}$	${\bar{Y}}_{i}=\beta_{0}+\beta_{1}{\bar{X}}_{i}$
Ordenada en el origen (*β*_0_). (IC 95%)	0,0666 (entre −0,5626 y 0,6959)	−3,582 (entre −4,710 y −2,454)
Pendiente (*β*_1_). (IC 95%s)	1,0257 (0,9444 to 1,107)	1,4825 (entre 1,337 y 1,628)
**B) Prueba de F**	618,2	401,8
DFn	208,0	208,0
Valor P	<0,0001	<0,0001
Desviación de cero?	Significativa	Significativa
Número total de valores	210	210

**Figura 2:**
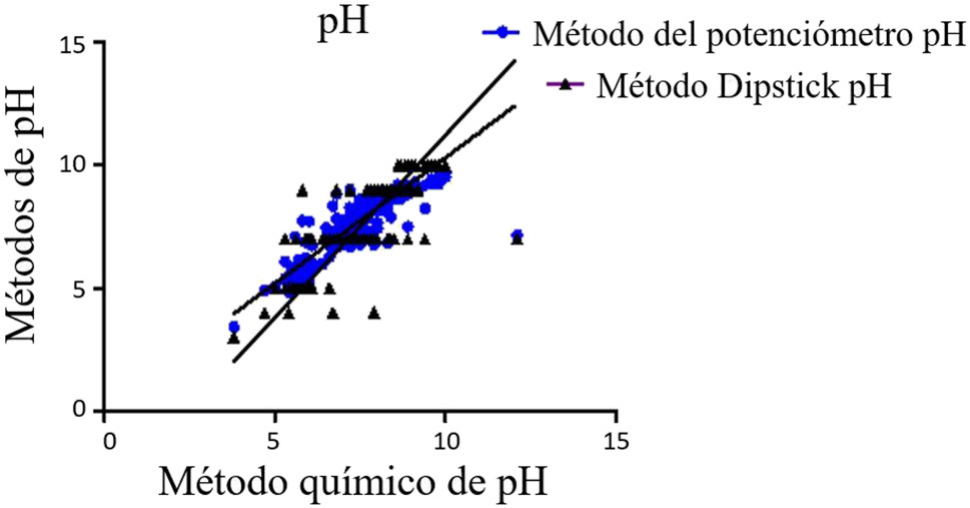
Análisis de regresión de Deming del método químico de análisis de Ph de Thermo Fisher frente al potenciómetro y la tira de detección de adulterantes en orina de TECO (Dipstick).

Se observó una diferencia estadísticamente significativa entre el potenciómetro y el método Dipstick, y el método de Thermo Fisher (Figura 2 y Tabla 1). El método Dipstick de determinación de pH mostró un mayor sesgo proporcional y constante que el potenciómetro, comparado con el método de Thermo Fisher.

Así mismo, las disoluciones estándar empleadas para la calibración del potenciómetro se analizaron con el método de Thermo Fisher empleando el analizador Beckman-Coulter AU5810, coincidiendo los resultados con las concentraciones de pH de los calibradores de los potenciómetros.

El Dipstick de Techo ha sido diseñado para su uso en métodos de química seca, ofreciendo resultados cuantitativos de pH y SG. El pH se basa en el método doble indicador de pH. La SG se basa en la variación de PKa de una serie de polielectrolitos pretratados, en relación con las concentraciones iónicas.

Para evaluar la precisión de los métodos de análisis de SG, pH, y oxidantes de Thermo Fisher en el analizador Beckman-Coulter, se realizaron medidas repetidas dos veces al día durante 20 días de las muestras de control y calibración. Los resultados para el refractómetro digital de Micro Palm Abbe y el medidor de pH/iones Mettler Toledo Seven Compact™ se muestran en la Tabla 2.

**Tabla 2:** Resultados de precisión para (A) Thermo Fisher pH. (B) Thermo Fisher pH. (C) Thermo Fisher Oxidant. (D) GE empleando el refratómetro digital Micro Palm Abbe. (E) pH empleando el medidor de pH/iones Mettler Toledo Seven Compact™.

A) Resultados de precisión del análisis de gravedad específica de Thermo Fisher. n=80			
Controles	Control 1	Calibrador de GE alta	Control 2
Media, g/mL	1,014	1,025	1,031
SD intraserial, g/mL	1,015 ± 0,0004	1,020 ± 0,0002	1,032 ± 0,0002
CV intraserial, %	0,04	0,05	0,06
SD interserial, g/mL	1,015 ± 0,0005	1,020 ± 0,0004	1,032 ± 0,0004
Interserial CV, %	0,04	0,05	0,06

B) Resultados de precisión para el análisis de pH de Thermo Fisher. n=60				
Controles	Control 1	Control 2	Control 3	Control 4
Media	3,6	7,0	10,0	11,5
SD intraserial	3,45 ± 0,1	7,18 ± 0,1	10,64 ± 0,1	11,50 ± 0,1
CV intraserial, %	3,6	1,2	1,0	1,0
SD interserial	3,45 ± 0,2	7,18 ± 0,1	10,64 ± 0,1	11,50 ± 0,2
Total CV, %	4,5	2,0	1,1	1,4

C) Resultados de precisión para el análisis de oxidantes de Thermo Fisher. n=120			
Controles	Control 1	Calibrador de GE alta	Control 2
Media, µg/mL	100	200	300
SD intraserial, µg/mL	84,8 ± 2,1	199,3 ± 2,9	322,1 ± 3,8
CV intraserial, %	2,4	1,5	1,2
SD interserial, µg/mL	84,8 ± 2,7	199,3 ± 3,6	322,1 ± 5,8
Interserial CV, %	3,2	1,8	1,8

D) Resultados de precisión para el refractrómetro digital Micro Palm Abbe PA202 para la GE. n=20			
Controles	Control 1	Calibrador de GE alta	Control 2
Media, g/mL	1,014	1,025	1,031
SD intraserial, g/mL	1,020 ± 0,0004	1,028 ± 0,0002	1,032 ± 0,0002
CV intraserial, %	0,05	0,05	0,06
SD interserial, g/mL	1,015 ± 0,0005	1,020 ± 0,0004	1,032 ± 0,0004
Interserial CV, %	0,05	0,05	0,06

E) Resultados de precisión para el medidor de pH/iones Mettler Toledo Seven Compact™ S220. n=20				
Controles	Control 1	Control 2	Control 3	Control 4
Media	3,6	7,0	10,0	11,5
SD intraserial, pH	3,50 ± 0,1	7,19 ± 0,1	10,66 ± 0,1	11,56 ± 0,1
CV intraserial, %	1,8	1,1	1,1	1,2
SD interserial, pH	3,45 ± 0,2	7,18 ± 0,1	10,64 ± 0,1	11,50 ± 0,2
Total CV, %	2,0	1,5	1,6	1,5

También se realizó un análisis de los resultados con respecto a los intervalos de referencia, con el fin de realizar una evaluación desde un punto de vista clínico y sintetizar el resultado global para los diferentes métodos. Basándonos en el intervalo de referencia de 1,003–1,035 para la SG, se observó una concordancia del 81,9% entre el método de Thermo Fisher en el analizador Beckman-Coulter AU5810 y el refractómetro. La concordancia entre el refractómetro y Dipstick fue del 66,1%. La concordancia entre el analizador Beckman-Coulter AU5810, los métodos de análisis de SG y Dipstick fue del 64,7% (Tabla 3).

Aplicando un intervalo de referencia de 4,5–8,0 para el pH, se observó una concordancia del 74,3% entre el método de Thermo Fisher en el analizador Beckman-Coulter AU5810 y el refractómetro. La concordancia entre el pHímetro y Dipstick fue del 85,7%. El método de Thermo Fisher en el analizador Beckman-Coulter AU5810 y Dipstick mostraron una concordancia del 81,4% (Tabla 3).

**Tabla 3:** Comparación de los métodos de análisis de GE y pH y concordancia con respecto al intervalo de referencia.

Comparación de distintos métodos de análisis de GE con respecto al intervalo de referencia (IR) 1.003–1.035	GE (Analizador químico Beckman-Coulter AU5810)	GE (Refractómetro)	GE Tira de detección de adulterantes en orina TECO (Dipstick)
N° total de análisis	204	204	204
N° de resultados dentro del intervalo de referencia	197	173	129
N° de resultados por debajo del IR	6	19	52
N° de resultados por encima del IR	1	12	23
G.E. Concordancia entre el análisis químico de Thermo Fisher y la refractometría		167 de 204 (81,9%)	
G.E. Concordancia entre la refractometría y la Tira de detección de adulterantes en orina TECO (Dipstick)		135 de 204 (66,1%)	
G.E.Concordancia entre el análisis químico de Thermo Fisher y la Tira de detección de adulterantes en orina TECO (Dipstick)		132 de 204 (64,7 %)	
**Comparación de distintos métodos de análisis de pH con respecto al intervalo de referencia (IR) 4,5–8,0**	**pH (Analizador químico Beckman-Coulter AU5810 ser)**	**pH (Potenciómetro)**	**pH (Tira de detección de adulterantes en orina TECO (Dipstick))**

N° total de análisis	210	210	210
N° de resultados por debajo del IR	1	1	5
N° de resultados por encima del IR	84	128	102
Análisis de pH. Concordancia entre el análisis químico de Thermo Fisher y el potenciómetro		156 de 210 (74,3%)	
Análisis de pH. Concordancia entre el potenciómetro y la Tira de detección de adulterantes en orina TECO (Dipstick))		180 de 210 (85,7%)	
Análisis de pH. Concordancia entre el análisis químico de Thermo Fisher y la Tira de detección de adulterantes en orina TECO (Dipstick))		171 de 210 (81,4%)	

En la Tabla 4 se muestran los resultados obtenidos sobre interferencias en las diluciones seriadas para clorocromato de piridinio, nitrito de sodio grado de laboratorio, material de referencia certificado de nitrito, nitrato de sodio e hipoclorito de sodio (lejía) mediante el análisis de oxidantes y Dipstick en los días 1 y 17. La concentración mínima de clorocromato de piridinio (25 μg/mL) fue negativa en el día 17. Las concentraciones de hipoclorito de sodio se deterioraron con el tiempo, por la reducción de la estabilidad [11]. El Dipstick de Techo ofrece resultados semicuantitativos para el nitrito, el clorocromato de piridinio y el hipoclorito de sodio.

**Tabla 4:** Resultados obtenidos en las diluciones en serie de Clorocromato de piridinio, nitrito de sodio de laboratorio, material de referencia certificado de nitrito, e hipoclorito de sodio (Lejía) medidos mediante el análisis de oxidantes y la tira reactiva de adulterantes UrineCheck 7.

Clorocromato de piridinio	Análisis de oxidantes, µg/mL		Tira de detección de adulterantes en orina TECO-clorocromato de piridinio (PC)		
	Día 1	Día 17	Día 1	Día 17	Creatinina mmol/L
PC 400, µg/mL	674	537	Positivo	Positivo	100
PC 200, µg/mL	352	281	Positivo	Positivo	100
PC 100, µg/mL	185	140	Positivo	Positivo	100
PC 50, µg/mL	92	74	Positivo	Positivo	100
PC 25, µg/mL	47	32	Positivo	Negativo	100
Orina limpia	0	0	0	0	100

**Nitrito de sodio (de laboratorio)**	**Análisis de oxidantes, µg/mL**		**Tira de detección de adulterantes en orina TECO- nitrito**		
	**Día 1**	**Día 17**	**Día 1**	**Día 17**	**Creatinina mmol/L**

Nitrito 400, µg/mL	328	317	Positivo (>15 mg/dL o >150 μg/mL)		100
Nitrito 200, µg/mL	146	141	Positivo (>15 mg/dL o >150 μg/mL)		100
Nitrito 100, µg/mL	59	63	Positivo (>15 mg/dL o >150 μg/mL)		100
Nitrito 50, µg/mL	28	27	Positivo (0,5–5 mg/dL o 5–50 μg/mL)		100
Nitrito 25, µg/mL	12	11	Positivo (0,5–5 mg/dL o 5–50 μg/mL)		100
Orina limpia	0	0	0		100

**Nitrito (Material de referencia certificado)**	**Análisis de oxidantes, µg/mL**		**Tira de detección de adulterantes en orina TECO (nitrito)**		
	**Día 1**	**Día 17**	**Día 1**	**Día 17**	**Creatinina mmol/L**

Nitrito 1000, µg/mL	1157	1156	Positivo (>15 mg/dL o >150 μg/mL)		100
Nitrito 500, µg/mL	542	543	Positivo (>15 mg/dL o >150 μg/mL)		100
Nitrito 250, µg/mL	259	257	Positivo (>15 mg/dL o >150 μg/mL)		100
Nitrito 125, µg/mL	122	123	Positivo (0.5–5 mg/dL o 5–50 μg/mL)		100
Nitrito 62.5, µg/mL	58	59	Positivo (0.5–5 mg/dL o 5–50 μg/mL)		100
Orina limpia	0	0	0		100

**Nitrato de sodio (de laboratorio)**	**Análisis de oxidantes, µg/mL**		**Tira de detección de adulterantes en orina TECO(nitrato)**		
	**Día 1**	**Día 17**	**Día 1**	**Día 17**	**Creatinina mmol/L**

Nitrato 400, µg/mL	0		0		100
Nitrato 200, µg/mL	0		0		100
Nitrato 100, µg/mL	0		0		100
Nitrato 50, µg/mL	0		0		100
Nitrato 25, µg/mL	0		0		100
Orina limpia	0		0		100

**Hipoclorito de sodio (Lejía)**	**Análisis de oxidantes, µg/mL**		**Tira de detección de adulterantes en orina TECO (hipoclorito de sodio)**		
	**Día 1**	**Día 17**	**Día 1**	**Día 17**	**Creatinina mmol/L**

Lejía 4%	1058	135	Positivo	Positivo	100
Lejía 2%	455	13	Positivo	Negativo	100
Lejía 1%	149	2	Positivo	Negativo	100
Lejía 0.5%	42	0.7	Positivo	Negativo	100
Orina limpia	0	0	Negativo	Negativo	100

## Discusión

La SG es la medición de la densidad de un líquido con respecto a la densidad del agua y mide la concentración de partículas disueltas en la muestra. Un valor bajo de SG puede deberse a una excesiva ingesta de líquidos, fallo renal, diabetes insípida, entre otros factores. Un valor elevado de SG puede estar relacionado con la deshidratación, disfunción renal, y otros factores médicos como la secreción de la hormona antidiurética desencadenada por el estrés, un traumatismo y algunas drogas.

La refractometría mide el índice refractivo, que está relacionado con la masa total de solutos presentes en la orina. Las sustancias con un alto peso molecular como la glucosa, las proteínas, o los medios de contraste radiológicos tendrán un mayor efecto en la SG. Por otro lado, las tiras reactivas miden la fuerza iónica y no se ven afectadas por las proteínas, la glucosa o los medios de contraste. La osmolalidad, que mide la SG indirectamente, se ve afectada por la glucosa, pero no por los medios de contraste [12]. Existe evidencia de que la orina patológica muestra una peor correlación entre la SG y la osmolalidad que la orina "limpia". Las variables que afectan a la correlación son el pH, las cetonas, la bilirrubina, el urobilinógeno, la glucosa y las proteínas en la tira reactiva, y las cetonas, la bilirrubina y la hemoglobina en la refractometría [13].

El hecho de que las distribuciones de datos para el método Dipstick en la regresión de Deming parezcan mostrar una distribución horizontal en algunas partes se puede deber a que el método Dipstick tiene un rango de medición cuantitativa limitado para el pH y la SG. Así mismo, el método Dipstick no proporciona los decimales de pH, lo cual representa una limitación.

En un estudio se compararon los valores de SG obtenidos con Clinitek Dipstick, un refractómetro y un osmómetro. Los valores del refractómetro y el osmómetro mostraron una buena correlación lineal. El método Dipstick no resultó fiable para la SG, incluso tras la corrección del pH, tal como recomienda el fabricante [14]. Sin embargo, en otro estudio se observó una concordancia entre el método Dipstick y el refractómetro [15]. Dicha inconsistencia se puede deber a que en el otro estudio se observaron diferencias en los valores de SG entre los diferentes refractómetros. Dichas diferencias dependían del refractómetro, y los resultados de un instrumento podrían afectar a las decisiones clínicas [16].

El índice de refracción de una disolución varía con la suma de todos los sólidos disueltos en dicha disolución. La relación entre el índice de refracción y la SG en orina o la concentración proteica no es la misma en todos los refractómetros, así como tampoco lo es la metodología empleada en las mediciones [17].

El nivel de pH determina la acidez o alcalinidad de la muestra. Aunque algunas patologías afectan al pH de la orina, el valor de referencia fisiológico es de entre 4,5 y 9,0. El pH de una muestra de orina puede elevarse hasta 9,5, debido a condiciones de almacenamiento inadecuadas como una temperatura elevada. El ascorbato interfirió negativamente en los valores de hemoglobina, glucosa y nitrito. El ácido acetilsalicílico redujo el pH, siendo el efecto aún mayor en ausencia de la proteína [18].

El pH de la orina está directamente relacionado con la alimentación. Algunos alimentos con elevado nivel de acidez son los cereales, el pescado, los refrescos, los alimentos ricos en proteínas y los alimentos azucarados.

Algunos alimentos alcalinos son las nueces, las verduras y la mayoría de las frutas. Un pH elevado en orina (orina alcalina), podría indicar una patología como cálculos renales, infecciones del tracto urinario y diversas patologías renales. Los vómitos prolongados en el tiempo también pueden provocar que la orina tenga un pH elevado. Esto previene la acidez estomacal, lo que puede hacer que los fluidos corporales sean más alcalinos.

La orina ácida también puede favorecer la formación de cálculos. Una orina con un pH bajo, (orina ácida) podría indicar la presencia de una patología como la cetoacidosis diabética, una complicación de la diabetes, diarrea y malnutrición. Algunos fármacos también pueden afectar al pH de la orina. En la noche o la mañana anterior al análisis de orina, se deberán suspender algunos fármacos, salvo que se quiera determinar el pH de la orina en relación con un fármaco concreto.

Si el valor de alguno de estos parámetros queda fuera de los rangos de normalidad, la muestra podría haber sido adulterada.

Actualmente se ofrecen en el mercado diferentes adulterantes oxidantes, bajo el reclamo de que eliminan todos los resultados positivos de las pruebas de detección de drogas.

Algunos adulterantes oxidantes son el nitrito (Klear™), el cromato (Urine Luck™), el yodo, la lejía y la peroxidasa de rábano picante/H_2_O_2_ (Stealth™). Cuando se añaden a la orina no varía el aspecto de la misma, ni su pH, SG o concentración de creatinina. Las muestras de orina de los sujetos que han consumido marihuana adulteradas con oxidantes pueden dar un resultado positivo en una prueba inicial mediante inmunoensayo, especialmente el tetrahidrocannabinol (THC), un metabolito de la marihuana. Sin embargo, estos no se pueden confirmar mediante GC-MS o LC-MS [19, 20].

El análisis de oxidantes se puede realizar en analizadores automáticos para detectar la presencia de oxidantes. Este método se basa en la reacción entre la tetrametilbencidina (TMB) y el oxidante presente en la muestra, produciendo un color que se puede medir a 660 nm. Algunos oxidantes como el nitrito se pueden generar en el organismo y excretarse a través de la orina mediante la oxidación enzimática por el óxido nítrico sintasa (NOS). Sin embargo, la mayor parte del nitrito resultante se oxida y convierte en nitrato. Por tanto, la concentración de nitrato en la orina humana resultante de la actividad de la NOS es mucho mayor que la concentración de nitrito. Un estudio realizado en voluntarios sanos reveló una concentración media de nitrato de 61 μg/mL y de nitrito de 0,2 μg/mL en orina [21]. Los pacientes con infección del tracto urinario o con algunas enfermedades pueden presentar concentraciones de nitrito de hasta 100 y 150 μg/mL. Las muestras de orina a las que se añadió Klear™ como fuente de nitrito contenían concentraciones de nitrito de entre 1.900 y 15.000 μg/mL. De este modo, una concentración de nitrito en orina de 200 μg/mL es una prueba científicamente válida y sólida en términos forenses de adulteración de la muestra con una sustancia con contenido de nitrato. También se encuentra cromato en el organismo, aunque en concentraciones muy bajas. Las concentraciones normales de cromo en orina varían entre 0,04 y 1,0 μg/mL [21, 22].

## Conclusiones

El método de análisis de SG de Thermo Fisher mostró mejor concordancia con el refractómetro que con la tira de detección de adulterantes de TECO. El método de pH de Thermo Fisher mostró una mayor concordancia con la tira reactiva de TECO que con el potenciómetro. A la luz de los resultados obtenidos y de su mejor concordancia, precisión y mejor tiempo de respuesta, recomendamos el empleo de los métodos químicos automáticos para medir la creatinina, y el análisis de oxidantes, pH y SG para realizar el estudio de validez de la muestra. Si un análisis de oxidantes en orina es negativo y los resultados de pH y/o SG no están en el rango de normalidad, se recomienda analizar dicha muestra de orina sospechosa con Axiom assay™ de Axiom Diagnostics Incorporated, para comprobar si se trata de orina sintética.
